# Integrated single-cell sequencing of 5-hydroxymethylcytosine and genomic DNA using scH&G-seq

**DOI:** 10.1016/j.xpro.2021.101016

**Published:** 2021-12-11

**Authors:** Alex Chialastri, Chatarin Wangsanuwat, Siddharth S. Dey

**Affiliations:** 1 Department of Chemical Engineering, University of California Santa Barbara, Santa Barbara, CA 93106, USA; 2 Center for Bioengineering, University of California Santa Barbara, Santa Barbara, CA 93106, USA; 3 Neuroscience Research Institute, University of California Santa Barbara, Santa Barbara, CA 93106, USA

**Keywords:** Bioinformatics, Cell isolation, Single Cell, Genomics, Sequencing, Molecular Biology

## Abstract

The asymmetric distribution of 5-hydroxymethylcytosine (5hmC) between two DNA strands of a chromosome enables endogenous reconstruction of cellular lineages at an individual-cell-division resolution. Further, when integrated with data on genomic variants to infer clonal lineages, this combinatorial information accurately reconstructs larger lineage trees. Here, we provide a detailed protocol for single-cell 5-hydroxymethylcytosine and genomic DNA sequencing (scH&G-seq) to simultaneously quantify 5hmC and genomic DNA from the same cell to reconstruct lineage trees at a single-cell-division resolution.

For complete details on the use and execution of this protocol, please refer to Wangsanuwat et al., 2021.

## Highlights

• scH&G-seq enables quantification of 5hmC and genomic DNA (gDNA) from the same cell
• Strand-specific 5hmC enables reconstructing trees at a single-cell-division resolution
• Genomic variants allow identification of clonal subtrees within a lineage tree
• Integrated 5hmC and gDNA detection improves prediction accuracy of larger lineage trees

## Graphical abstract

**Figure:**
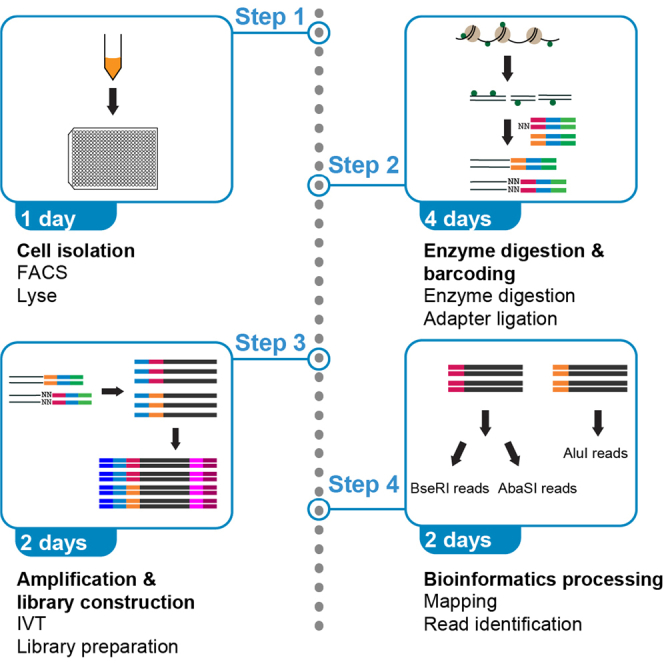

## Before you begin

This protocol for scH&G-seq (Single-cell 5-hydroxymethylcytosine and genomic DNA sequencing) was recently developed in a publication to simultaneously quantify 5hmC and genomic DNA from a single cell (Wangsanuwat et al., 2021). scH&G-seq builds upon scAba-Seq (Mooijman et al., 2016), where the addition of restriction enzymes BseRI and/or AluI enables sequencing of genomic DNA together with 5hmC in single cells. BseRI was selected because it generates the same overhang after digestion of genomic DNA as AbaSI, which is used to detect 5hmC. In addition, the recognition sequence of BseRI is devoid of CpG, eliminating ambiguity in assigning reads to genomic DNA or 5hmC. Thus, both genomic DNA-derived reads and 5hmC-derived reads can be separated computationally post-sequencing while being ligated and amplified from the same double-stranded adapter. AluI was selected to increase the coverage of the genome, as described previously (Rooijers et al., 2019). As digestion with AluI leaves a blunt end, an additional blunt double-stranded adapter is required to capture genomic DNA reads derived from AluI. The AluI double-stranded adapters and AbaSI/BseRI double-stranded adapters use different sets of unique barcodes. This protocol provides step-by-step instructions for performing scH&G-seq using both BseRI and AluI simultaneously. It also includes modifications to the protocol if only one of the two enzymes are used. Finally, scH&G-seq can be performed on any cell type that can be sorted by fluorescence-activated cell sorting (FACS) and has 5hmC in its genome.

The scH&G-seq protocol is completed over several days. Day 1 in the table below is the day a sorted 384-well plate containing single cells is thawed from −80°C for use in the scH&G-seq protocol. Use the table below to plan your experimental timeline.

**Table:** 

Protocol steps	Performed on day
Restriction digestion of genomic DNA	1
Stripping chromatin from genomic DNA	1
Glucosylation of 5hmC sites in the genome	2
Protease treatment to degrade T4-BGT	3
AbaSI digestion	4
Ligating double-stranded adapters to fragmented genomic DNA molecules	4
Pooling single cells and DNA cleanup	5
*In vitro* transcription and amplified RNA clean-up	5 & 6
Illumina library preparation from amplified RNA	6
Computational pipeline to analyze scH&G-seq sequencing	7 and beyond

Before starting the experiment, prepare double-stranded adapters, add Vapor-Lock to 384-well plates, and culture the cells of interest.

### Prepare double-stranded adapters

**Timing: 6 h**

The barcoded double-stranded adapters used for ligation in this protocol are generated by phosphorylating one of the DNA strands followed by the annealing of two single-stranded DNA molecules: top and bottom strand primer. The double-stranded adapters are then diluted down to their working concentration. All pipetting steps here are performed manually.

1. Phosphorylate the bottom strand of each barcoded adapter (for both AbaSI/BseRI and AluI)
  a. Perform reaction in a 96-well plate. Keep each barcoded bottom strand primer in a separate well.

**Table:** 

Phosphorylation mix	Final concentration	Volume
T4 Ligase buffer (10×)	1×	2 μL
ATP (10 μM)	0.5 μM	1 μL
T4 Polynucleotide Kinase (10,000 units/mL)	1.25 units/μL	2.5 μL
Nuclease-free water	n/a	4.5 μL
Barcoded bottom strand primer (100 μM)	50 μM	10 μL
**Total**	**n/a**	**20 μL**
  b. Seal the plate with a Greiner multiwell plate seal, and briefly spin the plate down at 2500 rpm. Then incubate as follows:

**Table:** 

Thermocycler conditions			
Steps	Temperature	Time	Cycles
DNA phosphorylation	37°C	1 h	1
Heat inactivation	65°C	20 min	1
Final hold	4°C	Forever	

Maintain thermocycler lid at 75°C. The total volume per well will be 20 μL.

2. Anneal top and bottom strand primers to generate double-stranded adapters.
  a. Perform this step in a 96-well plate. Add a top strand primer to each well containing the corresponding bottom strand primer that was phosphorylated in step 1.
  b. Before removing the seal from the plate, allow the plate to cool to 4°C in the thermocycler, then briefly spin the plate down at 2500 rpm.
  c. Add to step 1:

**Table:** 

Annealing mix	Final concentration	Volume
T4 Ligase buffer (10×)	1×	3 μL
Nuclease-free water	n/a	17 μL
Barcoded top strand primer (100 μM)	20 μM	10 μL
**Total**	**n/a**	**30 μL**
  d. As described in the table below, the thermocycler is preheated to 95°C, and once it has reached this temperature, insert the plate and advance to the next step. Incubate the plate for 5 min at 95°C, then decrease temperature by 1°C per minute till the plate reaches 20°C (Maintain thermocycler lid at 105°C). The total volume per well will be 50 μL.

**Table:** 

Thermocycler conditions			
Steps	Temperature	Time	Cycles
Preheating	95°C	Forever (advance to next step when the plate is inserted into the thermocycler)	
Initial denaturation	95°C	5 min	1
Gradual cooling	−1°C from previous	1 min	74
Final hold	20°C	Forever
  e. The barcoded double-stranded adapters are now double-stranded and are at a stock concentration of 20 μM.

3. Dilute the double-stranded adapters to a concentration of 1 μM.
  a. In a new 96-well plate, for each barcoded double-stranded adapter, combine 5 μL of the 20 μM stock (step 2) with 95 μL of nuclease-free water. The total volume per well will be 100 μL. This 96-well plate now contains 1 μM phosphorylated double-stranded adapters.
4. Further dilute the double-stranded adapters. Take the 1 μM phosphorylated double-stranded adapters from step 3 and follow the steps below to dilute them to their corresponding working concentrations in a total volume of 100 μL per well.
  a. For all scH&G-seq strategies, the working concentration of the random 2-nucleotide 3′ overhang phosphorylated double-stranded adapters (that is, for ligating to AbaSI/BseRI generated fragments) is 75 nM. Make a new 96-well plate for this working concentration. To do this, for each barcoded double-stranded adapter, combine 7.5 μL of 1 μM stock (step 3) with 92.5 μL of nuclease-free water. The total volume per well will be 100 μL. The adapter sequences have been described in detail previously (Mooijman et al., 2016).
  b. The working concentration of the blunt phosphorylated double-stranded adapters (that is, for ligating to AluI generated fragments) is 64 nM. Make a new 96-well plate for this working concentration. To do this, for each barcoded double-stranded adapter, combine 6.4 μL of 1 μM stock (step 3) with 93.6 μL of nuclease-free water. The total volume per well will be 100 μL. The adapter sequences have been described in detail previously (Rooijers et al., 2019; Markodimitraki et al., 2020).
5. Seal all the plates with a Greiner multiwell plate seal, and briefly spin the plates down at 2500 rpm.

**CRITICAL:** When preparing phosphorylated double-stranded adapters, it is critical that each barcoded adapter remains separate and that they are not accidentally mixed or contaminated. Always use new tips for all steps. To avoid errors, it is recommended that barcode 1 go in well A1, barcode 2 in A2, barcode 3 in A3…etc. of the 96-well plate.

***Note:*** To expedite the above process, use a 12-channel pipette when handling the barcoded double-stranded adapters and for making dilutions. Mix the solution well by pipetting up and down at least 10 times at each stage. All primers and double-stranded adapters can be stored long term at −20°C.

***Note:*** The bottom strand primer refers to the primer that will ligate to the free 3′ end of genomic DNA after enzymatic digestion. The top strand primer refers to the primer that will ligate to the free 5′ end of genomic DNA after enzymatic digestion.

### Add Vapor-Lock to 384-well plates

**Timing: 10 min per plate**

To avoid evaporation of small volumes of aqueous solutions added in later steps, Vapor-Lock is added to each well of a 384-well plate. All pipetting at this step is performed manually.

6. Add 4 μL of Vapor-Lock to each well of a 384-well plate.
  a. A 12-channel pipette can be used for dispensing. To do this, use a serological pipette to dispense Vapor-Lock into a disposable pipetting reservoir. Typically, 5.5 mL of Vapor-Lock is needed to make 3 plates.
7. Seal the plate with a Greiner multiwell plate seal and briefly spin the plate down at 2500 rpm.

**CRITICAL:** Avoid touching the tips containing Vapor-Lock onto the top of the plate as this will result in a small amount of oil on the surface of the plate, considerably reducing the ability of the Greiner multiwell plate seal to effectively seal the plate.

***Note:*** The same pipette tips can be used to fill 2 plates with Vapor-Lock. Use new tips if any of the tips touch any other surface that could be contaminated with RNases.

***Note:*** PCR safe mineral oil can be used as a cheaper alternative to Vapor-Lock. The Vapor-Lock or mineral oil is added manually to avoid introducing oil into the liquid-handling robot. In this protocol, we prepared double-stranded adapters in 96-well plates and Vapor-Lock in 384-well plates, but scH&G-seq can be performed in just 96-well or 384-well plates.

### Culture H9 cell line

**Timing: 2+ weeks**

The cells of interest are grown under the desired experimental conditions, and when ready they are isolated for scH&G-seq. As an example here, H9 human embryonic stem cells are grown.

8. On ice, dilute the Matrigel in DMEM/F-12 according to the lot specific dilution factor. Then precoat a 6-well dish with the diluted Matrigel (1 mL/well). Incubate the plate at 20°C–25°C for 1 h for same day use or wrap in parafilm and store at 4°C for up to 1 week. Aspirate off superfluous liquid before plating cells.
9. Thaw a vial containing 1 million H9 cells. Put 70% of the cells into one Matrigel coated well and the other 30% into another Matrigel coated well of a 6-well dish.
  a. Over the next few days continue culturing the well that looks healthier and grows at the appropriate rate. Healthy colonies have smooth edges and are round, containing closely packed cells with a high nucleus to cytoplasm ratio.
10. Fill each well with 2 mL of pre-warmed mTeSR1.
11. Refresh the media daily and wash with 1× DPBS if cell debris is visible.
12. When cells reach approximately 70% confluency (this should take 5–7 days), and before colonies begin to combine, passage the cells using the cell dissociation reagent Versene.
  a. Aspirate the media and wash with 1× DPBS
  b. Add 1 mL of Versene to each well that requires passage.
  c. Incubate the plate at 37°C for 3–5 min.
  d. Aspirate Versene gently. The colonies should still be attached to the plate and should be visible to the unaided eye.
  e. Use a P1000 pipette to slowly add cell culture media onto the colonies, removing them from the plate. Do not use the pipette tip to scratch the colonies off the plate.
  f. Pipette up and down to create clumps of cells. Be gentle to prevent a single-cell suspension. During pipetting, avoid the formation of air bubbles.
  g. Passage the cells in small clumps. Typically, one well at approximately 70% confluency can be passed into 6–12 new Matrigel coated wells.
  h. Add up to 2 mL of media per well and disperse the clumps of cells evenly throughout the well.
13. Continue to grow the cells for at least one more passage, or until the cells are ready for single cell collection by FACS according to your experimental procedure. For H9 cells grown here, cells are ready for FACS sorting when they reach approximately 70% confluency and before colonies begin to combine.

**CRITICAL:** Pipetting with the appropriate amount of force to create small clumps of H9 cells is critical. Creating a single-cell suspension of H9 cells during passage will result in substantial cell death. Passaging in large clumps will result in the new plate quickly becoming overgrown. When initially attempting the protocol, it is recommended to err on the side of passaging cells in larger clumps and checking cell growth often.

***Note:*** Replace the cell culture steps as appropriate. It is critical that the resulting cells can be sorted by FACS and that the cells contain detectable levels of 5hmC.

## Key resources table

**Table:** 

REAGENT or RESOURCE	SOURCE	IDENTIFIER
**Chemicals, peptides, and recombinant proteins**		

TrypLE Select Enzyme (1×), no phenol red	Thermo Fisher Scientific	12563011
Fetal Bovine Serum	Thermo Fisher Scientific	10437028
Vapor-Lock	Qiagen	981611
T4 Polynucleotide Kinase	New England BioLabs	M0201S
Propidium iodide solution	Sigma-Aldrich	P4864-10ML
FACS test tube with a cell strainer snap cap	Fisher Scientific	08-771-23
Deoxynucleotide (dNTP) Solution Mix	New England BioLabs	N0447L
NEBuffer 4 (10×)	New England BioLabs	B7004S
Qiagen Protease	Qiagen	19155
T4 Phage β-glucosyltransferase (T4-BGT)	New England BioLabs	M0357L
AbaSI	New England BioLabs	R0665S
T4 DNA Ligase	New England BioLabs	M0202M
Adenosine 5′-Triphosphate	New England BioLabs	P0756L
Agencourt AMPure XP	Beckman Coulter	A63880
Agencourt RNAClean XP	Beckman Coulter	A63987
SuperScript II Reverse Transcriptase	Thermo Fisher Scientific	18064014
RNaseOUT Recombinant Ribonuclease Inhibitor	Thermo Fisher Scientific	10777019
NEBNext High-Fidelity 2× PCR Master Mix	New England BioLabs	M0541L
IGEPAL CA-630	Sigma-Aldrich	I8896-50ML
Recombinant Ribonuclease Inhibitor	Clontech	2313A
AluI	New England BioLabs	R0137S
BseRI	New England BioLabs	R0581S
Potassium acetate solution	Sigma-Aldrich	95843-100ML-F
Trizma acetate (Tris-acetate)	Sigma-Aldrich	93337-25G
Magnesium acetate solution	Sigma-Aldrich	63052-100ML
Ethylenediaminetetraacetic acid solution	Sigma-Aldrich	03690-100ML
Gibco DPBS, no calcium, no magnesium	Thermo Fisher Scientific	14-190-144
mTeSR1	Stemcell Technologies	85850
Gibco Versene solution	Thermo Fisher Scientific	15-040-066
Corning Matrigel hESC-qualified matrix	Thermo Fisher Scientific	08-774-552
DMEM/F-12, GlutaMAX supplement	Thermo Fisher Scientific	10565018
Nuclease-free Water (not DEPC-Treated)	Thermo Fisher Scientific	4387936
Absolute Ethanol, 200 proof, Molecular Biology Grade	Thermo Fisher Scientific	T038181000

**Critical commercial assays**		

MEGAscript T7 Transcription Kit	Thermo Fisher Scientific	AMB13345
Bioanalyzer High Sensitivity DNA Analysis	Agilent	5067-4626
Bioanalyzer High Sensitivity RNA Analysis (RNA 6000 Pico)	Agilent	5067-1513
Qubit Assay Tubes	Thermo Fisher Scientific	Q32856
Qubit dsDNA HS Assay Kit	Thermo Fisher Scientific	Q32854

**Deposited data**		

scH&G-seq sequencing data of H9 cells	Wangsanuwat et al., 2021	GEO: GSE131678

**Experimental models: Organisms/strains**		

Human: H9	WiCell	WA09

**Oligonucleotides**		

AbaSI adapters (Random 2-nucleotide 3′ overhang double-stranded adapters)	Mooijman et al., 2016	N/A
Random hexamer primer (for aRNA reverse transcription)	Hashimshony et al., 2016	N/A
Illumina sequencing primers	Hashimshony et al., 2016	N/A
AluI adapters (blunt end double-stranded adapters)	Rooijers et al., 2019	N/A
TruSeq Small RNA PCR Primers (RNA PCR Primers, RP1 and RPI1-RPI48)	Integrated DNA Technologies	Published by Illumina

**Software and algorithms**		

scPECLR (MATLAB)	Wangsanuwat et al., 2021	N/A
scH&G-seq analysis	This Paper	https://github.com/alexchialastri/scH-G-seq
Burrows-Wheeler Aligner (BWA)	Li and Durbin (2009)	Version 0.7.15
Perl	perl.org	Version 5.10.1
Linux operating system	N/A	N/A
Human genome	UCSC Genome Browser	hg19

**Other**		

Nanodrop II (low volume liquid handling robot)	BioNex Solutions	N/A
2100 Bioanalyzer instrument	Agilent	G2939BA
Qubit 2.0 Fluorometer	Thermo Fisher Scientific	Q32866
Sony SH800 cell sorter	Sony Biotechnology	SH800S
Centrifuge 5810R (refrigerated and plate compatible)	Eppendorf	2231000771
Vacufuge plus	Eppendorf	022820109
Greiner multiwell plate sealers	Sigma-Aldrich	Z617601-100EA
RNaseZap RNase Decontamination Solution	Thermo Fisher Scientific	AM9782
C1000 Touch Thermal Cycler with 384-Well Reaction Module	Bio-Rad Laboratories	1851138
Eppendorf 5331 MasterCycler Gradient Thermal Cycler	Eppendorf	5331
VWR 96-Well PCR and Real Time PCR Plates	VWR	82006-704
Hard-Shell 384-Well PCR Plates, thin wall, skirted, clear/clear	Bio-Rad Laboratories	HSP3801
Thermo Scientific BioLite Multidishes and Microwell Plate	Fisher Scientific	12-556-004
PR Series Analytical Balance	Ohaus	PR124
DynaMag-2 Magnet (Magnetic Stand)	Thermo Fisher Scientific	12321D
Disposable Pipetting Reservoirs	VWR	89094-664
Pipetman M Multichannel P12x20M, 1–20 μL	Gilson	F81028
10 μL-XL Aerosol barrier, low retention pipet tips	VWR	10017-062
20 μL Aerosol barrier, low retention pipet tips	VWR	10017-066
300 μL Aerosol barrier, low retention pipet tips	VWR	10017-088
1250 μL Aerosol barrier, low retention pipet tips	VWR	10017-092
Low-profile 0.2 mL 8-tube strips without caps (used with nanodrop II liquid handling robot)	Bio-Rad Laboratories	TLS0801
0.2 mL strip tubes (12 tubes per strip)	Fisher Scientific	AB-1112

## Materials and equipment

For high-throughput processing, the Nanodrop II low-volume liquid-handling robot was used unless otherwise noted. For dispensing reagents into the 384-well plate, the “dispense plate discrete stop” program built into the Nanodrop II robot was used. When dispensing reagents, 100 μL (12.5 μL per dispensing tip) of additional master mix is needed to account for the dead volume in the dispensing reservoir. For dispensing double-stranded adapters, the “dispense plate in-the-well” program built into the Nanodrop II robot was used, and between each dispense step the tips were washed three times with the pre-programed “Eject Wash Rinse” program to prevent inadvertently mixing barcoded double-stranded adapters.

***Note:*** Any liquid-handling robot with the ability to dispense into 384-well plates down to 200 nL volumes can be used. For manual pipetting, aerosol barrier, low retention pipet tips should be used to reduce losses and the possibility of cross contamination. Additionally, handling all materials in a PCR workstation can also reduce the chances of cross contamination. Finally, clean all surfaces and items (including gloves) using 70% ethanol followed by RNaseZap to reduce the chances of cross contamination.

**Table:** aRNA fragmentation buffer

Fragmentation buffer	Final concentration	Volume
Tris-acetate (0.5 M, pH 8.1)	200 mM	4 mL
Potassium acetate (5 M)	500 mM	1 mL
Magnesium acetate (1 M)	150 mM	1.5 mL
Nuclease-free water	n/a	3.5 mL
**Total**	**n/a**	**10 mL**

Store at 20°C–25°C indefinitely.

***Note:*** To ensure that the aRNA fragmentation buffer remains RNase-free, split the Tris-acetate into two parts. Use a pH probe to measure the exact amount of hydrochloric acid or sodium hydroxide required to balance the pH of one of the Tris-acetate aliquots to 8.1. Discard this aliquot, and to the other aliquot, add the measured amount of acid or base. Do not use the pH probe on this aliquot as it could result in RNase contamination.

## Step-by-step method details

**Figure 1:**
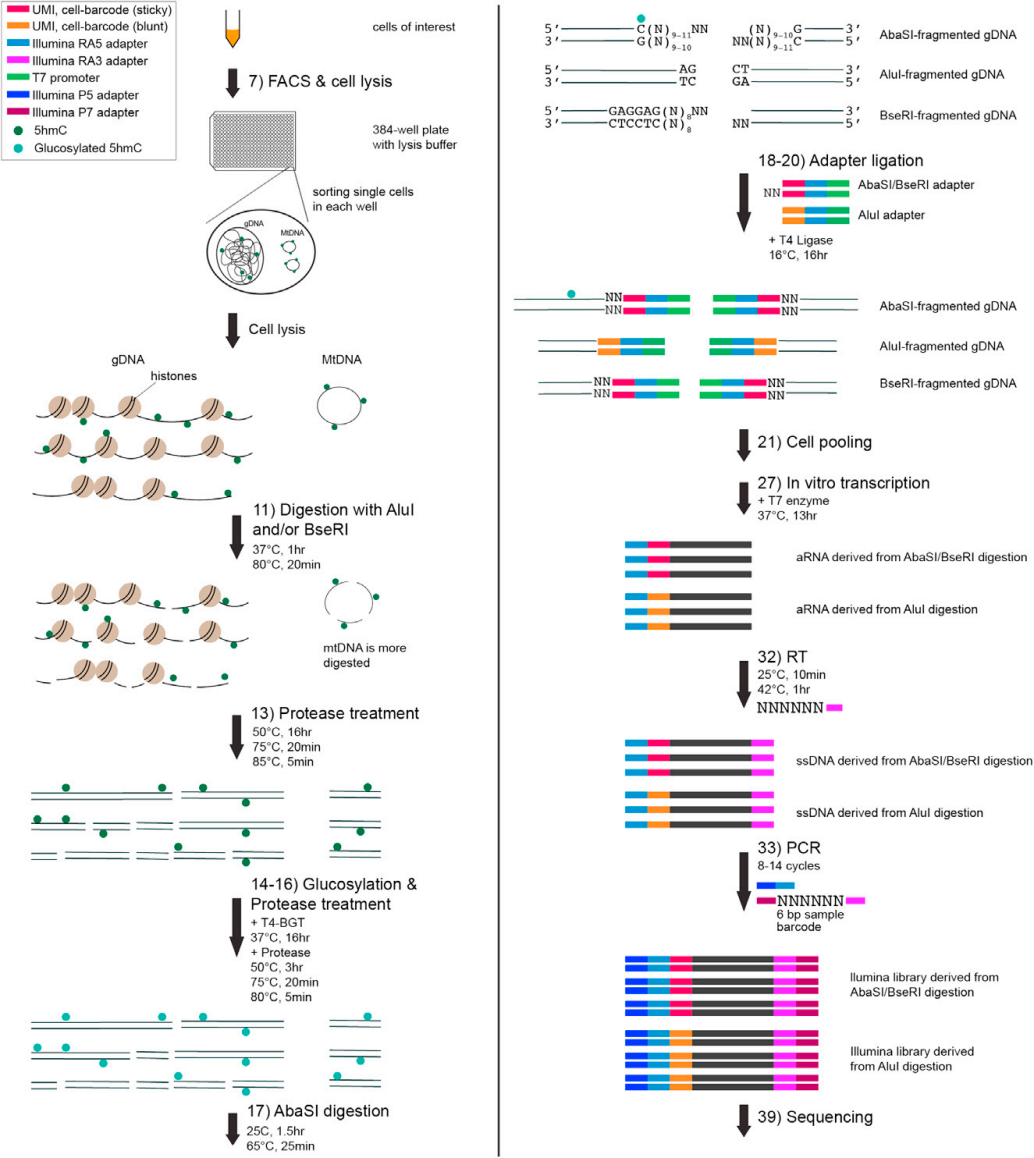
Schematic of scH&G-seq In scH&G-seq, cells of interest are first sorted into 384-well plates containing the lysis buffer. Next, AluI and/or BseRI are added to digest genomic and mitochondrial DNA. Following this step, protease treatment is used to strip off chromatin. Thereafter, 5hmC marks in the genome are glucosylated by T4-BGT and then protease treatment is used to degrade T4-BGT. Genomic DNA marked by modified 5hmC sites are then cut by AbaSI, which is followed by ligation with AbaSI-/BseRI-specific and AluI-specific double-stranded adapters. Next, single cells are pooled and amplified by *in vitro* transcription. Finally, after reverse transcription (RT) and PCR amplification, the Illumina libraries are ready for sequencing.

In the protocol below, we provide details of performing scH&G-seq and the corresponding data processing steps to analyze the Illumina sequencing data (Figure 1).

***Note:*** In each reaction mix table, the final concentration for each reagent added is calculated based on its concentration in the final solution volume in the 384-well plate rather than its concentration in the mix being added.

### Prepare plates for FACS by adding lysis buffer

**Timing: 1–2 h**

Add a lysis buffer to each well of a 384-well plate. This step is performed prior to FACS to ensure that once single cells are sorted into this solution, they are lysed and the plate can be stored at −80°C until processed.

1. To each well of a Vapor-Lock containing 384-well plate, add the lysis buffer as described below using a liquid handling robot:

**Table:** 

Lysis buffer	Final concentration	Volume
IGEPAL CA-630 (1%)	0.0875%	17.5 nL
Recombinant RNase Inhibitor (40 Units/μL)	0.93 units/μL	4.67 nL
Nuclease-free water	n/a	177.83 nL
**Total**	**n/a**	**200 nL**

2. Seal the 384-well plates with a Greiner multiwell plate seal, and briefly spin the plate down at 2500 rpm. Always keep plates on ice except when directly handling them.

### Isolate and FACS sort single cells

**Timing: 2–6 h (depending on the number of plates sorted)**

To make a single cell suspension of H9 cells (or cells of interest) and to sort one cell into each well of a 384-well plate, proceed as follows:

3. Wash cells twice with 2 mL of 1× DPBS.
4. Add 0.5 mL of TrypLE select (1×) solution to one well of a 6-well plate and incubate the plate at 37°C until cells begin to lift (3–5 min).
5. Add serum-containing media to neutralize the TrypLE solution, collect the cells in a new 15 mL conical centrifuge tube and spin the tube at 300 *g* for 5 min to pellet the cells.
6. Aspirate the medium and resuspend the pellet in 1 mL of 1× DPBS. Make a single-cell suspension by gently triturating with a P1000 pipette. Always keep the tube on ice except when handling it.
  a. Add propidium iodide (PI) to distinguish between live and dead cells, thereby increasing the capture efficiency. The final concentration of PI should be between 1 and 50 μg/mL.
  b. Filter cells using a FACS test tube with a cell strainer snap cap to remove large cellular clumps. After filtration, sort cells from this test tube.
7. Set up the FACS machine to sort single cells into a 384-well plate.
  a. If you are unsure whether the microfluidics of the FACS machine was cleaned prior to use, clean the instrument with 10% bleach. For the Sony SH800 cell sorter, follow the Bleach Cleaning wizard in the sorter software.
  b. Set the sample area to 4°C and, if possible, set the collection area on the FACS machine to 4°C as well.
  c. Use an empty 384-well plate sealed with a Greiner multiwell plate seal to perform a mock sort. Do this by dispensing 50 droplets of sheath fluid onto the seal covering the plate. Check that the droplets are centered on the wells of the plate. If they are not, adjust the alignment and re-perform the mock sort. Perform this mock sort on one fourth of all wells spread across the plate to ensure that the plate is properly aligned.

**CRITICAL:** Accurate alignment of droplets on the 384-well plate is needed for successfully sorting single cells into the plate.

8. Once the 384-well plate is well-aligned, sort a single cell into each well of the 384-well plate. Identify single cells using forward scatter and backscatter (or side scatter depending on the sorter).
9. After sorting a plate, seal it with a Greiner multiwell plate seal, and briefly spin the plate down at 2500 rpm. If sorting multiple plates, place the sorted plate on ice (or transfer to −80°C freezer) and sort the next plate following the same protocol.

***Optional:*** Leave a few wells unsorted as a negative control. Sequencing results from these negative wells will behave similar to poorly sorted wells. If you get a significant number of sequencing reads that map to these negative wells, it indicates genomic DNA contamination.

***Note:*** mTeSR1 does not contain serum. If serum containing media is unavailable, neutralize the TrypLE solution with a 5% fetal bovine serum solution in 1× DPBS.

***Note:*** Gentle pipetting is important to make a single cell suspension but over pipetting can reduce cell viability and increase cellular debris.

***Note:*** Single cells have a smaller forward scatter area and width when compared to doublets, but a similar forward scatter height. Additionally, single cells display higher forward scatter and lower back scatter than cellular debris. A high proportion of your cells should be viable single cells. Viable cells should contain no emission from propidium iodide.

**Pause point:** After sorting, plates can be stored at −80°C until ready to continue. Stored plates have similar success in generating high-quality Illumina libraries, even for plates that have been stored for up to 1 year.

### Restriction digestion of genomic DNA

**Timing: 2 h**

Genomic DNA is digested with the enzyme(s) of choice to sequence the genome in scH&G-seq. Simultaneous digestion with both BseRI and AluI is described here, but the options for digesting with only one of the two enzymes are also discussed.

10. Incubate the plate for 5 min at 65°C and then return it to 4°C or ice.

11. Digest genomic DNA simultaneously with both BseRI and AluI by adding the following mix to each well of the 384-well plate using a liquid handling robot.
  a. BseRI and AluI digestion master mix:

**Table:** 

BseRI and AluI digestion	Final concentration	Volume
NEBuffer 4 (10×)	1×	70 nL
BseRI (5,000 units/mL)	0.18 units/μL	25 nL
AluI (10,000 units/mL)	0.18 units/μL	12.5 nL
Nuclease-free water	n/a	392.5 nL
**Total**	**n/a**	**500 nL**
  b. Seal the plate with a Greiner multiwell plate seal and briefly spin the plate down at 2500 rpm.
  c. Incubate in a thermocycler as follows:

**Table:** 

Thermocycler conditions			
Steps	Temperature	Time	Cycles
Genomic DNA digestion	37°C	1 h	1
Heat inactivation	80°C	20 min	1
Final hold	4°C	Forever
    The total volume per well will be 700 nL (excluding Vapor-Lock).
    ***Optional:*** If only BseRI is used, replace the digestion mix in step 11a with the following mix. Perform steps 11b and 11c as described above.

**Table:** 

BseRI digestion	Final concentration	Volume
NEBuffer 4 (10×)	1×	70 nL
BseRI (5,000 units/mL)	0.36 units/μL	50 nL
Nuclease-free water	n/a	380 nL
**Total**	**n/a**	**500 nL**

    ***Optional:*** If only AluI is used, replace the digestion mix in step 11a with the following mix. Perform steps 11b and 11c as described above.

**Table:** 

AluI digestion	Final concentration	Volume
NEBuffer 4 (10×)	1×	70 nL
AluI (10,000 units/mL)	0.36 units/μL	25 nL
Nuclease-free water	n/a	405 nL
**Total**	**n/a**	**500 nL**

    ***Optional:*** If the goal is to detect only 5hmC and no genomic DNA, replace the digestion mix in step 11a with the following mix. Perform step 11b as described above but skip step 11c.

**Table:** 

No genomic digestion	Final concentration	Volume
NEBuffer 4 (10×)	1×	70 nL
Nuclease-free water	n/a	430 nL
**Total**	**n/a**	**500 nL**

    ***Note:*** Digestion of genomic DNA here is performed prior to stripping off chromatin to maximize reads derived from mitochondrial DNA. Mitochondrial reads can be useful for clonal lineage reconstruction as regions of the mitochondrial genome have higher rates of mutation compared to genomic DNA (Wallace, 1994; Ludwig et al., 2019; Xu et al., 2019).

### Stripping chromatin from genomic DNA

**Timing: 1 day**

Protease is added to remove chromatin, enabling the genome-wide detection of 5hmC.

12. If no Qiagen protease solution is available, starting from the lyophilized powder make a fresh Qiagen protease solution at a concentration of 100 μg/μL in nuclease-free water.

**CRITICAL:** Make the Qiagen protease solution under RNase-free conditions. A sterile pipette tip can be used instead of a traditional spatula to scoop out the lyophilized powder, while maintaining RNase-free conditions.

***Note:*** Qiagen protease solution can be stored at −20°C for up to one month.

13. Add the following mix to each well of the 384-well plate using a liquid handling robot.
  a. Protease master mix:

**Table:** 

Protease I	Final concentration	Volume
NEBuffer 4 (10×)	1×	180 nL
Qiagen Protease (100 μg/μL)	2.4 μg/μL	60 nL
Nuclease-free water	n/a	1,560 nL
**Total**	**n/a**	**1.8 μL**
  b. Seal the plate with a Greiner multiwell plate seal and briefly spin the plate down at 2500 rpm.
  c. Perform the following:

**Table:** 

Thermocycler conditions			
Steps	Temperature	Time	Cycles
Protease digestion	50°C	16 h	1
Heat inactivation I	75°C	20 min	1
Heat inactivation II	80°C	5 min	1
Final hold	4°C	forever
  d. The total volume per well will be 2.5 μL.
    **Pause point:** Plates can be stored for 12 h at 4°C.

### Glucosylation of 5hmC sites in the genome

**Timing: 1 day**

AbaSI has approximately 1,000 times more affinity for detecting 5hmC compared to 5-methylcytosine (5mC), but it has nearly 10,000 times greater affinity towards detecting beta-glucosyl-5-hydroxymethylated cytosine (5ghmC) compared to 5mC (Wang et al., 2011). Therefore, to significantly improve the specificity of 5hmC detection, T4 Phage β-glucosyltransferase (T4-BGT) is added to convert 5hmC sites in the genome to 5ghmC.

14. Convert 5hmC to 5ghmC by adding the following mix to each well of the 384-well plate using a liquid handling robot.
  a. BGT master mix:

**Table:** 

BGT	Final concentration	Volume
NEBuffer 4 (10×)	1×	50 nL
UDP-Glucose (50×)	1×	60 nL
T4-BGT (10,000 units/mL)	0.33 units/μL	100 nL
Nuclease-free water	n/a	290 nL
**Total**	**n/a**	**500 nL**
  b. Seal the plate with a Greiner multiwell plate seal and briefly spin down the plate at 2500 rpm.
  c. Perform the following:

**Table:** 

Thermocycler conditions			
Steps	Temperature	Time	Cycles
Glucosylation	37°C	16 h	1
Final hold	4°C	Forever
  d. The total volume per well will be 3 μL.
    **Pause point:** Plates can be stored for 12 h at 4°C.

### Protease treatment to degrade T4-BGT

**Timing: 5 h**

Protease is added to the reaction mixture to degrade T4-BGT.

15. If no Qiagen protease solution is available, make a fresh Qiagen protease solution at 100 μg/μL concentration in nuclease-free water as described in step 12.
16. Degrade T4-BGT by adding the following mix to each well of the 384-well plate using a liquid handling robot.
  a. Protease master mix:

**Table:** 

Protease II	Final concentration	Volume
NEBuffer 4 (10×)	1×	50 nL
Qiagen protease (100 μg/μL)	0.57 μg/μL	20 nL
Nuclease-free water	n/a	430 nL
**Total**	**n/a**	**500 nL**
  b. Seal the plate with a Greiner multiwell plate seal and briefly spin the plate down at 2500 rpm.
  c. Perform the following:

**Table:** 

Thermocycler conditions			
Steps	Temperature	Time	Cycles
Protease digestion	50°C	3 h	1
Heat inactivation I	75°C	20 min	1
Heat inactivation II	80°C	5 min	1
Final hold	4°C	Forever
  d. The total volume per well will be 3.5 μL.
    **Pause point:** Plates can be stored for 12 h at 4°C.

### AbaSI digestion

**Timing: 3 h**

AbaSI is added to selectively cut genomic DNA downstream of 5hmC and 5ghmC sites, leaving a 2-nucleotide 3′ overhang.

17. Cut genomic DNA downstream of 5hmC and 5ghmC sites by adding the following mix to each well of the 384-well plate using a liquid handling robot.
  a. AbaSI digestion master mix:

**Table:** 

AbaSI digestion	Final concentration	Volume
NEBuffer 4 (10×)	1×	50 nL
AbaSI (10,000 units/mL)	0.25 units/μL	100 nL
Nuclease-free water	n/a	350 nL
**Total**	**n/a**	**500 nL**
  b. Seal the plate with a Greiner multiwell plate seal and briefly spin the plate down at 2500 rpm.
  c. Perform the following:

**Table:** 

Thermocycler conditions			
Steps	Temperature	Time	Cycles
AbaSI digestion	25°C	1.5 h	1
Heat inactivation	65°C	25 min	1
Final hold	4°C	forever
  d. The total volume per well will be 4 μL.

### Ligating double-stranded adapters to fragmented genomic DNA molecules

**Timing: 1 day**

The double-stranded adapters are ligated to genomic DNA fragments cut by AluI, BseRI, and AbaSI. These adapters are specific for each enzyme and uniquely barcoded so that single cells can be pooled downstream in the protocol. The adapters also contain a part of the Illumina read 1 adapter sequence and a T7 promoter sequence.

18. Add 200 nL of the 64 nM blunt phosphorylated double-stranded adapter to each well of the 384-well plate using a liquid handling robot. Use one unique adapter per well (Figure 2).
  a. After adding the double-stranded adapter, seal the plate with a Greiner multiwell plate seal and briefly spin the plate down at 2500 rpm.

**Figure 2:**
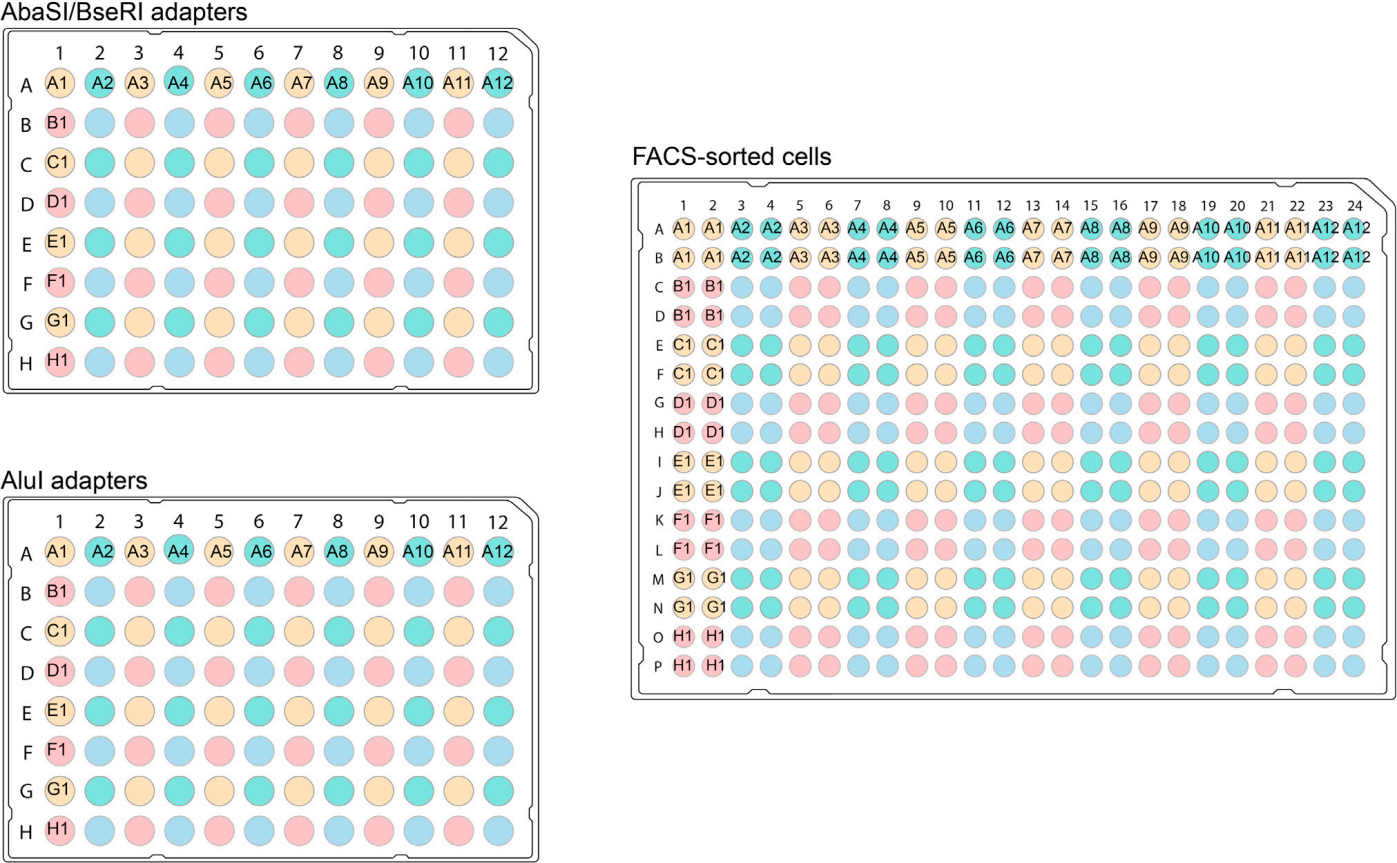
Dispensing double-stranded adapters from 96-well plates to a 384-well plate Both AbasI-/BseRI-specific double-stranded adapters and AluI-specific double-stranded adapters are dispensed from 96-well plates to a 384-well plate containing cells of interest. The double-stranded adapter in well A1 of the 96-well plate is dispensed into wells A1, A2, B1, and B2 of the 384-well plate. Similarly, the double-stranded adapter in well A2 of the 96-well plate is dispensed into wells A3, A4, B3, and B4 of the 384-well plate and so on. The color scheme in the figure above indicates how double-stranded adapters in 96-well plates are dispensed into a 384-well plate containing single cells. After ligation, the single cells in the 384-well plate are pooled into four microcentrifuge tubes, with each tube containing 96 single cells with distinct barcodes.
19. Add 200 nL of the 75 nM random 2-nucleotide 3′ overhang phosphorylated double-stranded adapter to each well of the 384-well plate using a liquid handling robot. Use one unique adapter per well (Figure 2).
  a. After adding the double-stranded adapter, seal the plate with a Greiner multiwell plate seal and briefly spin the plate down at 2500 rpm.
20. Ligate the adapters to the fragmented genomic DNA molecules by adding the following mix to each well of the 384-well plate using a liquid handling robot.
  a. T4 DNA ligase master mix:

**Table:** 

Ligation	Final concentration	Volume
T4 DNA ligase buffer (10×)	0.2×	100 nL
T4 DNA ligase (2,000,000 units/mL)	28 units/μL	70 nL
ATP (10 mM)	0.8 mM	400 nL
Nuclease-free water	n/a	30 nL
**Total**	**n/a**	**600 nL**
  b. Seal the plate with a Greiner multiwell plate seal and briefly spin the plate down at 2500 rpm.
  c. Perform the following:

**Table:** 

Thermocycler conditions			
Steps	Temperature	Time	Cycles
Ligation	16°C	16 h	1
Final hold	4°C	Forever
  d. The total volume per well will be 5 μL. The combined concentration of NEBuffer 4 and T4 DNA ligase buffer is 1×.
    **CRITICAL:** The cells that you want to pool together must have uniquely barcoded double-stranded adapters added at this step. Do not add more than one barcoded double-stranded adapter of each adapter type to a single reaction well.
    **CRITICAL:** Wash the tips of the robot three times with the pre-programed “Eject Wash Rinse” program after each adapter dispenses to prevent inadvertently mixing the double-stranded adapters.
    **CRITICAL:** Document the double-stranded adapters used for each cell. 5hmC derived (from AbaSI) and BseRI derived genomic DNA reads will have the same barcode for a given cell, while AluI derived genomic DNA reads will have another set of barcodes. AbaSI/BseRI barcodes and AluI barcodes for the same cell will later be matched during data processing. Without this documentation, it will not be possible to pair the 5hmC derived reads, and the BseRI and AluI derived reads to the same cell.
    ***Note:*** ATP and the T4 DNA ligase buffer, which contains ATP, degrade with multiple freeze thaw cycles. Aliquot these components as necessary to limit the number of freeze thaw cycles.
    ***Optional:*** If AluI is not used to digest genomic DNA, do not add the blunt phosphorylated double-stranded adapters (skip step 18). In addition, replace the ligation mix (step 20a) with the following mix shown below. Thereafter, perform steps 20b and 20c as described above.

**Table:** 

Ligation	Final concentration	Volume
T4 DNA ligase buffer (10×)	0.2×	100 nL
T4 DNA ligase (2,000,000 units/mL)	28 units/μL	70 nL
ATP (10 mM)	0.8 mM	400 nL
Nuclease-free water	n/a	230 nL
**Total**	**n/a**	**800 nL**

    **Pause point:** Plates can be stored at −20°C until ready to continue.

### Pooling single cells and DNA cleanup

**Timing: 3 h**

Each well containing distinctly barcoded DNA molecules from an individual cell in the 384-well plate is pooled together manually. A standard 1× DNA bead cleanup is performed to exchange the buffer.

21. Use a manual pipette (a 12-channel P20 is recommended) to pool cells that are uniquely barcoded (both AbaSI/BseRI barcodes and AluI barcodes) into a 1.5 mL microcentrifuge tube. Each well of the 384-well plate should contain a total volume of 9 μL (4 μL Vapor-Lock and 5 μL of aqueous solution) (Figure 2).
  a. Depending on the numbers of AbaSI/BseRI and AluI barcodes used, more than one 1.5 mL microcentrifuge tube might be required to pool cells from the entire plate. For example, if 96 AbaSI/BseRI barcodes and 96 AluI barcodes were distributed over a 384-well plate, four 1.5 mL microcentrifuge tubes will be required to pool cells.
  b. To pool a plate formatted as described in Figure 2, for the first barcoded set of 96 cells, pool all odd rows and odd columns. Use a 12-channel P20 pipette to aspirate all liquid from wells A1, A3, A5…A23, then eject the liquid into a 12-tube PCR strip.
  c. Return to the plate and aspirate wells C1, C3, C5…C23, then eject the liquid into the same 12-tube PCR strip.
  d. Continue this pattern until the odd columns of row O have been transferred into the same 12-tube PCR strip. The same tips can be used throughout this process but should be discarded after transferring the odd columns of row O.
  e. Transfer the material from the 12-tube PCR strip into a 1.5 mL microcentrifuge tube. The pooling of one set of 96 uniquely barcoded cells are now complete.
  f. Repeat this process using new tips, a new 12-tube PCR strip and a new 1.5 mL microcentrifuge tube to pool all even rows and odd columns. This will result in the second set of 96 uniquely barcoded cells.
  g. Repeat this process using new tips, a new 12-tube PCR strip and a new 1.5 mL microcentrifuge tube to pool all odd rows and even columns. This will result in the third set of 96 uniquely barcoded cells.
  h. Repeat this process again using new tips, a new 12-tube PCR strip and a new 1.5 mL microcentrifuge tube to pool all even rows and even columns. This will result in the fourth set of 96 uniquely barcoded cells. At this point the 384-well plate has been distributed into 4 libraries and each library has its own 1.5 mL microcentrifuge tube.
22. Spin the tubes at 5,000 *g* for 1 min. The mixture should separate into two distinct phases.
23. Use a P200 pipette to remove as much of the upper oil phase as possible.
24. Next, use a P1000 pipette to quickly plunge through any of the remaining oil phase and transfer the aqueous phase to a new tube.
  a. The pipette should go through the oil phase only once to collect all the solution in the aqueous phase. If the oil and aqueous phases begin to mix prior to complete separation of the aqueous phase, repeat steps 22–24.
  b. The samples are not amplified at this point. To minimize sample loss, collect as much of the aqueous layer as possible, and ideally leave behind less than 10 μL of the aqueous phase. It is best practice to not aspirate any of the oil phase as it can complicate step 25.

**CRITICAL:** Minimize transferring the oil phase during the phase separation. The presence of oil makes the DNA bead cleanup step more challenging as the beads become hydrophobic and do not dry easily. While the presence of oil does not affect the quality of the final Illumina libraries, it can increase handling time during the bead cleanup step. Therefore, if some oil is still present in the aqueous phase after the transfer in step 24, repeat steps 22–24 to remove the oil.

25. Measure the volume transferred to the new tubes with a P1000 pipette and perform a 1× AMPure XP magnetic bead cleanup by adding the same volume of the magnetic bead solution as the sample.
  ***Note:*** Prior to use, allow the magnetic bead solution to reach 20°C–25°C and vortex the magnetic bead solution well.
  a. Mix the beads and sample thoroughly and then incubate at 20°C–25°C for 30 min.
  b. Move the tube to a magnetic stand. Wait till the beads form a pellet.
  c. Discard the supernatant.
  d. Wash the pellet twice with freshly made (up to 7 days) 80% ethanol, ensuring that it covers the entire bead pellet.
  e. While still leaving the tube on the magnetic stand, allow the pellet to dry with the tube’s lid left open for approximately 3–10 min. The beads are dry when a slight color change appears. Do not over-dry the beads. Once minor cracking of the beads is visible, move to the next step.
  f. Remove the tube from the magnetic stand and elute DNA with 30 μL of nuclease-free water.
  g. Mix well and wait 15 min before placing the tube on the magnetic stand.
  h. After the beads form a pellet, transfer the supernatant to a new tube. The supernatant now contains the unamplified DNA molecules.
    **CRITICAL:** The samples have not been amplified at this point, so minimizing material loss is critical during the elution step (step 25.h). Try to transfer at least 29 μL in step 25.h.

26. Use a vacufuge at 20°C–25°C to reduce the sample volume from 30 μL to 6.4 μL.
  a. If the sample does not reduce to 6.4 μL, see troubleshooting 1.
    **CRITICAL:** Each 1.5 mL microcentrifuge tube should contain pooled DNA molecules from individual cells with unique AbaSI/BseRI barcodes and AluI barcodes. For example, if 96 cells are pooled into the same 1.5 mL microcentrifuge tube, 96 unique AbaSI/BseRI barcodes and 96 unique AluI barcodes are required.
    ***Note:*** When using a vacufuge, if the sample volume reduces to less than 6.4 μL, add nuclease-free water to bring the volume back up to 6.4 μL.
    **Pause point:** Samples can be stored at −20°C until ready to continue.

### *In vitro* transcription and amplified RNA clean-up

**Timing: 1 day**

*In vitro* transcription (IVT) is used to amplify the barcoded DNA molecules, generating amplified RNA (aRNA). Next, ExoSAP-IT is used to remove any excess primers. The aRNA is then fragmented to a suitable length (200–1000 nucleotides) for sequencing. Finally, a standard 0.825× RNA bead cleanup is performed to exchange the buffer.

27. The MEGAscript T7 transcription kit is used for this step. To each pooled sample containing 6.4 μL of barcoded DNA molecules add:
  a. IVT master mix:

**Table:** 

IVT (MEGAscript T7 transcription kit)	Final concentration	Volume
ATP solution	n/a	1.6 μL
CTP solution	n/a	1.6 μL
GTP solution	n/a	1.6 μL
UTP solution	n/a	1.6 μL
T7 reaction buffer (10×)	1×	1.6 μL
T7 enzyme mix	n/a	1.6 μL
**Total**	**n/a**	**9.6 μL**
  b. The total volume per sample will be 16 μL containing 1× T7 reaction buffer.
  c. Perform the following:

**Table:** 

Thermocycler conditions			
Steps	Temperature	Time	Cycles
IVT	37°C	13 h	1
Final hold	4°C	forever
  d. The samples are now amplified and contain aRNA.
    **CRITICAL:** As IVT takes 13 h to complete, it is likely that this step will be performed at the end of the day and the samples will be held at 4°C. While aRNA is stable at 4°C for several hours, it is recommended to either store aRNA at −20°C for short-term storage (a few days) or proceed to the next step within a few hours after the IVT step is complete.

28. Add 6 μL of ExoSAP-IT and incubate the samples for 15 min at 37°C.
29. aRNA fragmentation
  a. Preheat a thermocycler to 94°C.
  b. Add 5.5 μL of fragmentation buffer to the samples while on ice.
  c. Immediately transfer the sample tubes to the thermocycler at 94°C for exactly 2 min.
  d. Immediately place sample tubes back on ice.
  e. Add 2.75 μL of 0.5 M EDTA to stop fragmentation.
  f. Add 19.75 μL of nuclease-free water. The total volume should now be 50 μL.

**CRITICAL:** Due to the time-sensitive nature of the aRNA fragmentation step, it is recommended to handle only 2–3 tubes at one time.

30. Briefly spin down the tubes and transfer the solution to a new 1.5 mL microcentrifuge tube.
31. Perform a standard 0.825× RNA bead cleanup, eluting in 22 μL of water.
  a. This step is similar to step 25.
  b. Add 41.25 μL of RNAClean XP magnetic beads to the fragmented aRNA. Incubate the sample for 15 min and then transfer to a magnetic stand.
  c. Once the beads form a pellet, discard the supernatant and wash the pellet twice with freshly made 80% ethanol.
  d. Dry the beads on the magnetic stand by leaving the cap of the tube open (3–10 min). Remove the tube from the magnetic stand and elute the fragmented aRNA in 22 μL of nuclease-free water.
  e. Allow the fragmented aRNA to elute from the beads for 10 min before placing the tube on the magnetic stand. Once the beads form a pellet, transfer the supernatant to a new tube.

**CRITICAL:** All surfaces and items must be RNase-free. Use RNaseZap to wipe down all equipment, surfaces, and gloved hands. RNase contamination can lead to library preparation failure.

**Figure 3:**
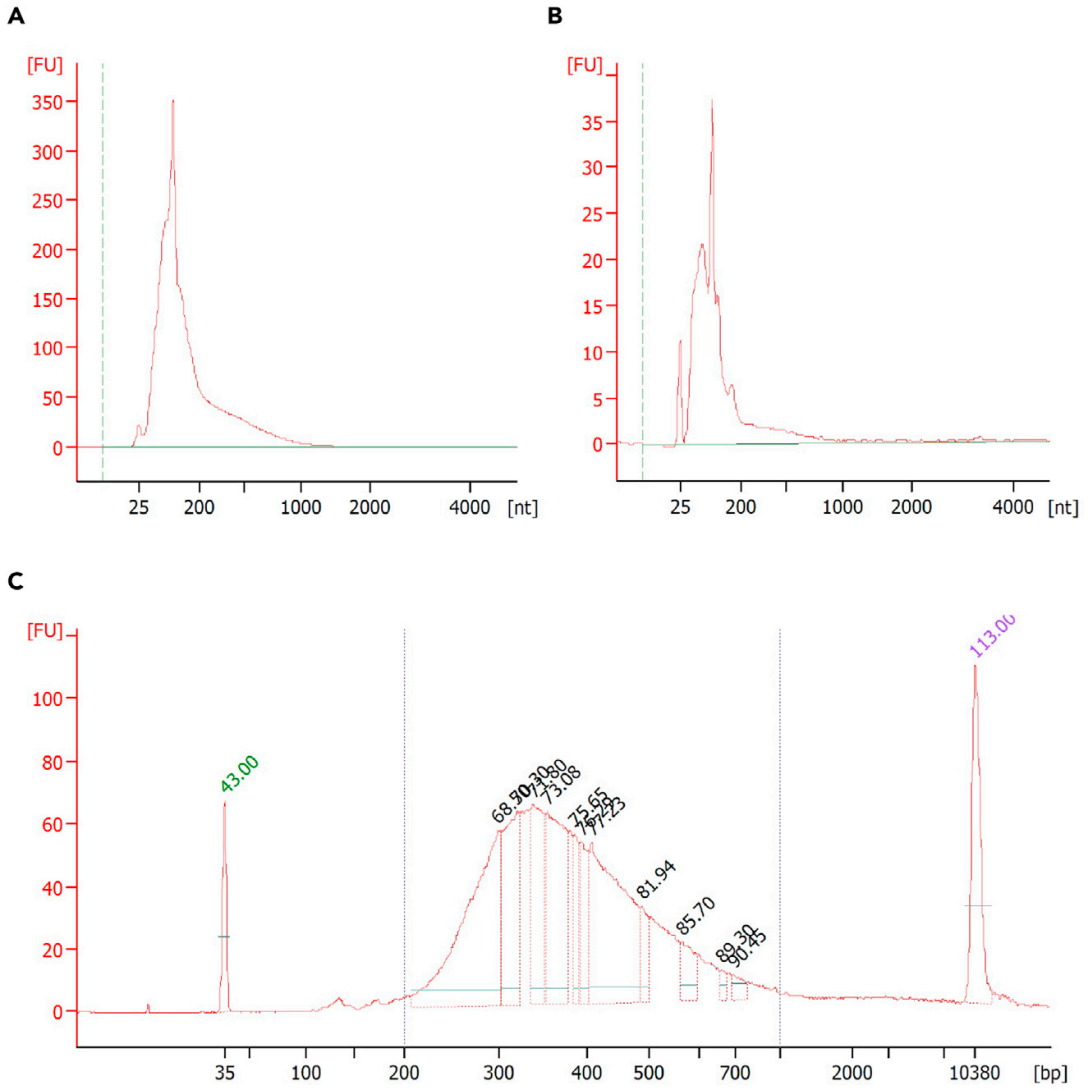
Expected size distribution of aRNA and Illumina library in scH&G-seq (A and B) Representative bioanalyzer plot for aRNA run on an Agilent RNA 6000 Pico chip. More material amplified RNA is typically observed when all three enzymes AbaSI, BseRI, and AluI are used (A), compared to when AbaSI is used alone (B). (C) Representative bioanalyzer plot for a scH&G-seq Illumina library run on an Agilent high-sensitivity DNA chip. A majority of the library should be within the 200–1000 base pair range. Fluorescence units ([FU]) on the y-axis correlates with the amount of RNA/DNA in the sample.

***Optional:*** At the completion of this section, an Agilent RNA 6000 Pico chip can be run to check the size distribution of the fragmented aRNA (Figures 3A and 3B).

**Pause point:** Samples can be stored at −80°C until ready to continue. In the following steps, not all aRNA will be used. The remaining aRNA can be stored at −80°C to generate additional Illumina libraries in the future if necessary.

### Illumina library preparation from amplified RNA

**Timing: 1 day**

In this section, aRNA is reverse transcribed using random hexamer primers with overhangs that contain part of the Illumina read 2 adapter sequence. The reverse transcribed single-stranded DNA now contains parts of the Illumina read 1 and read 2 adapters at the two ends of the molecules. Next, these molecules are amplified by PCR, and the full Illumina adapter sequences are introduced using overhangs in the PCR primers. Two 0.825× DNA bead cleanups are performed to remove primers and dimers, and to exchange the buffer. The size distribution and concentration of the final Illumina libraries are then quantified using an Agilent Bioanalyzer and Qubit fluorometer, respectively.

32. Reverse transcription of aRNA
  a. To 5 μL of the aRNA from step 31, add:

**Table:** 

Reverse transcription part 1	Final concentration	Volume
Random hexamer primer (20 μM)	3.07 μM	1 μL
dNTP (10 mM)	0.77 mM	0.5 μL
**Total**	**n/a**	**1.5 μL**
  b. The total volume will be 6.5 μL.
  c. Perform the following:

**Table:** 

Thermocycler conditions			
Steps	Temperature	Time	Cycles
Heat denature the aRNA	65°C	5 min	1
  d. Immediately place the samples directly on ice.
  e. Add the remaining reverse transcription mix:

**Table:** 

Reverse transcription part 2	Final concentration	Volume
First strand buffer (5×)	0.95×	2 μL
DTT (0.1 M)	9.52 mM	1 μL
RNaseOUT (40 Units/μL)	1.90 Units/μL	0.5 μL
SuperScript II (200 Units/μL)	9.52 Units/μL	0.5 μL
**Total**	**n/a**	**4 μL**
  f. The total volume will be 10.5 μL.
  g. Perform the following:

**Table:** 

Thermocycler conditions			
Steps	Temperature	Time	Cycles
Initial extension	25°C	10 min	1
Reverse transcription	42°C	1 h	1
Final hold	4°C	forever

33. PCR amplification
  a. Transfer 5.25 μL of the reverse transcribed aRNA from step 32 to a new PCR tube and store the other half at −20°C for later use in case the number of PCR cycles performed here turns out to be non-optimal.
  b. Each sample should receive its own indexed RNA PCR Primer (RPI1-RPI48) so that it can be multiplexed with other samples in one Illumina sequencing lane if desired.
  c. To 5.25 μL of the reverse transcribed aRNA, add the following PCR mix:

**Table:** 

PCR mix	Final concentration	Volume
RNA PCR primer (RP1) (10 μM)	0.40 μM	1 μL
RNA PCR primer (Choose one from RPI1-48) (10 μM)	0.40 μM	1 μL
NEBNext high-fidelity PCR mix (2×)	0.99×	12.5 μL
Nuclease-free water	n/a	5.5 μL
**Total**	**n/a**	**20 μL**
  d. The total volume is now 25.25 μL.
  e. Perform the following (Note that the thermocycler is preheated to 98°C, and once it has reached this temperature, insert the sample and advance to the next step):

**Table:** 

Thermocycler conditions			
Steps	Temperature	Time	Cycles
Preheating	98°C	Forever (advance to the next step when the PCR tubes are placed in the thermocycler)	
Initial denaturation	98°C	30 s	1
Denaturation	98°C	10 s	8–14 cycles
Annealing	60°C	30 s	
Extension	72°C	30 s	
Final extension	72°C	10 min	1
Hold	4°C	Forever

34. Briefly spin down the tubes to collect all the liquid to the bottom of the tube. Add 24.75 μL of nuclease-free water. Transfer the solution in each PCR tube to a new 1.5 mL microcentrifuge tube.
35. Perform a 0.825× AMPure XP magnetic bead cleanup, eluting in 50 μL of nuclease-free water.
  a. This cleanup is similar to the one described in step 25.
  b. Add 41.25 μL of AMPure XP magnetic beads to 50 μL of the PCR product. Incubate the sample for 15 min. Transfer the tube to a magnetic stand.
  c. Once the beads form a pellet, discard the supernatant. Wash the pellet twice with freshly made 80% ethanol.
  d. Dry the beads on the magnetic stand by leaving the cap of the tube open for 3–10 min. Remove the tube from the magnetic stand and elute in 50 μL of nuclease-free water.
  e. Allow the DNA to elute from the beads for 10 min before placing the tube on the magnetic stand. Once the beads form a pellet, transfer the supernatant to a new tube.
36. Perform a second 0.825× AMPure XP magnetic bead cleanup, eluting in 15 μL of nuclease-free water.
  a. This cleanup is the same as step 35, except that the final elution volume is 15 μL.
37. Determine the fragment size distribution of the Illumina library using an Agilent high-sensitivity DNA kit following the manufacturer’s recommendations. 1 μL of each sample is loaded on a high-sensitivity DNA chip.
  a. If the Illumina library does not show a size distribution similar to Figure 3C, see troubleshooting 2, 3, 4, and 5

**CRITICAL:** The bioanalyzer plot should show that greater than 90% of the product library has a size distribution between 200 and 1000 bp. There should be little to no primer (<100 bases) or dimer peaks (∼120 bases) present. If there is a substantial primer and/or dimer peaks present, or if a significant percentage of the library is greater than 1000 bp, see troubleshooting 2 and 3.

38. If the bioanalyzer results warrant continuation, quantify the concentration of the Illumina library using the Qubit dsDNA high-sensitivity assay by following the manufacturer’s recommendations. 1 μL of the Illumina library is used to estimate the concentration.
  a. If the sample concentration is above the lower limit of detection for the Qubit dsDNA high-sensitivity assay, the concentration is typically sufficient for Illumina sequencing.
  b. The concentration will vary depending on the number of PCR cycles, but concentrations are typically in the range of 0.5–4 ng/μL. Low Qubit concentrations might reflect that many of the cells in the library have failed to amplify and may not produce high-quality data.
39. Combine Illumina libraries with distinct multiplexed barcodes added during the PCR step (step 33). Sequence on an Illumina platform to a depth of approximately 0.5–1 million reads per cell. For the analysis pipeline presented below, sequencing should contain at least 76 cycles on read 1. No custom Illumina sequencing primers are required.

***Note:*** During PCR (step 33), one of the primers is universal (RP1) and is added to all samples. To multiplex multiple samples on the same Illumina sequencing lane, the other primer (RPI1, RPI2, … RPI48) is unique to each sample.

***Note:*** 9 PCR cycles were found to be sufficient for making successful Illumina libraries containing 48 H9 cells.

***Note:*** These Illumina libraries can be sequenced without any custom primers, and they can be multiplexed with any TruSeq-based Illumina sequencing library.

***Note:*** While the steps 25, 35, and 36 are for cleanup of DNA molecules, the cleanup in step 31 is for RNA molecules. Make sure to use the correct magnetic beads (AMPure XP *vs.* RNAClean XP) accordingly.

**Pause point:** Samples can be stored at −20°C after the reverse transcription or PCR step (prior to or after AMPure XP magnetic bead cleanup).

### Computational pipeline to analyze scH&G-seq sequencing results

**Timing: 1–2 days**

Sequencing results are processed into output files that provide 5hmC and genomic/mitochondrial DNA locations in each cell. Each read type – AbaSI, BseRI, and AluI derived reads – is partitioned into different files. Each file contains a list of unique locations detected (with duplicate reads removed) with the following format: cell barcode number, chromosome, mapped genomic location, and DNA strand information. The DNA strand information column is replaced with a unique molecule identifier (UMI) for the AluI derived file. All scripts and barcodes used can be found at GitHub https://github.com/alexchialastri/scH-G-seq. The associated scH&G-seq sequencing data for H9 cells can be found at GEO: GSE131678.

40. Process reads derived from AbaSI (5hmC) and BseRI (genomic/mitochondrial DNA). These reads have the same barcode (which is a different set from AluI derived reads) and are deconvoluted using the following steps:
  a. Sequenced reads (read 1) are trimmed to 76 bases.
    i. perl MakeFastqShorter.pl -Input InputFastqFileName.fastq -LengthMake 76
  b. Trimmed reads containing AbaSI/BseRI barcodes are extracted.
    i. perl ExtractingAbaReads_96BC_SimultanousWithAluI_UserInput.pl -START_DIR ./ -FASTQ_R1 InputFastqFileName-Trimed-76.fastq -BARCODES aba_barcodes.csv -ALUIBARCODES ALUI.txt
  c. Read 1 is then mapped using Burrows Wheels Aligner (BWA).
    i. bwa aln -q 0 -n 0.04 -k 2 -l 200 -t 6 -B 6 hg19.fa InputFastqFileName-Trimed-76-AbaSI-BseRI.fastq > Read1OutputFileName.sai
    ii. bwa samse -n 100 hg19.fa Read1OutputFileName.sai InputFastqFileName-Trimed-76-AbaSI-BseRI.fastq > Read1OutputFileName.sam
  d. The resulting mapped output sam file is then converted to the final processed AbaSI and BseRI files based on the recognition site of each restriction enzyme.
    i. perl process_scaba_with_BseRI_AnyInput.pl hg19.fa Read1OutputFileName.sam aba_barcodes.csv
    ii. perl Aba_or_BseRI_FabaToText.pl Read1OutputFileName-ABA.faba
    iii. perl Aba_or_BseRI_FabaToText.pl Read1OutputFileName-BseRI.faba
  e. The final output file Read1OutputFileName-ABA.txt contains 5hmC locations detected in single cells and Read1OutputFileName-BseRI.txt contains BseRI-derived genomic/mitochondrial DNA locations detected in the same single cells.
41. Deconvolute and process AluI-derived reads.
  a. Sequenced reads (read 1) are trimmed to 76 bases using the MakeFastqShorter.pl Perl script. Skip this step if step 40.a was performed.
    i. perl MakeFastqShorter.pl -Input InputFastqFileName.fastq -LengthMake 76
  b. Trimmed reads containing AluI barcodes are extracted.
    i. perl ExtractingAluIReads_UserInput_NoBarcodeCollisions.pl -FASTQ_R1 InputFastqFileName-Trimed-76.fastq -ALUI_BC ALUI.txt -ABASI_BC aba_barcodes.csv
  c. Read 1 is then mapped using Burrows Wheels Aligner (BWA).
    i. bwa aln -q 0 -n 0.04 -k 2 -l 200 -t 6 -B 13 hg19.fa InputFastqFileName-Trimed-76-AluI.fastq > Read1AluIOutputFileName.sai
    ii. bwa samse -n 100 hg19.fa Read1AluIOutputFileName.sai InputFastqFileName-Trimed-76-AluI.fastq > Read1AluIOutputFileName.sam
  d. The resulting mapped output sam file is then converted to the final processed AluI file based on its recognition site.
    i. perl Identifying_AluI_Sites_withUMI_SCdata_ForkedAdapters.pl Read1AluIOutputFileName.sam Read1AluIOutputFileName-se_MappedReads.txt Read1AluIOutputFileName-se_correctpos_stringent.txt
    ii. perl SimplifyData.pl Read1AluIOutputFileName-se_correctpos_stringent.txt Read1AluIOutputFileName-se_correctpos_stringent_simplified.txt ALUI.txt
    iii. sort -k1,1n -k2,2V -k3,3n Read1AluIOutputFileName-se_correctpos_stringent_simplified.txt > Read1AluIOutputFileName-se_correctpos_stringent_simplified_sort.txt
    iv. perl RemoveDup.pl Read1AluIOutputFileName-se_correctpos_stringent_simplified_sort.txt Read1AluIOutputFileName-se_correctpos_stringent_simplified_sort_rmdup.txt
  e. The final output file Read1AluIOutputFileName-se_correctpos_stringent_simplified_sort_rmdup.txt has AluI-derived genomic/mitochondrial DNA locations detected in single cells.
42. Note that AluI-derived reads and AbaSI/BseRI-derived reads have different barcodes. By keeping track of which AluI and AbaSI/BseRI barcodes were added to a given cell (noted in steps 18–20), the corresponding reads derived from AluI and AbaSI/BseRI can be assigned to the same cell.

***Note:*** Only single-end sequencing is required. Replace InputFastqFileName.fastq with the fastq file name from your experiment.

***Note:*** Prior to mapping, index the genome using BWA version 0.7.15. Follow the steps outlined in the BWA manual to create an indexed genome.

***Note:*** BWA parameters in steps 40.c and 41.c can be adjusted as appropriate. However, “-B” parameter indicating the barcode length should be kept as 6 and 13, respectively.

***Note:*** The names of output files can be adjusted as appropriate, as long as the names of files generated in steps 40 and 41 remain distinct.

***Note:*** For the pipeline processing BseRI and AbaSI derived reads, the resulting text files will indicate chromosome X as 23, chromosome Y as 24, and mitochondrial-based reads as chromosome 25. The following scripts will also work on the mouse genome (or any organism with less than 22 autosomes) and will still result in the same naming convention for the sex chromosomes and mitochondrial-based reads. Organisms with more than 22 autosomes will require slight modification to line 65 of process_scaba_with_BseRI_AnyInput.pl to account for more chromosomes. For the pipeline processing AluI derived reads, the resulting text files will identify the sex chromosomes as chromosome X and Y, and the mitochondrial-based reads as chromosome M.

***Optional:*** After mapping the fastq files, samtools can be used to quantify the mapping efficiency using the flagstat tool on both sam files. Samtools can also be used to convert the sam file into a compressed bam file for long term data storage.

***Optional:*** After completing this protocol, follow the steps described for single-cell Probabilistic Endogenous Cellular Lineage Reconstruction (scPECLR) in Wangsanuwat et al., 2021 to reconstruct cellular lineages.

## Expected outcomes

**Figure 4:**
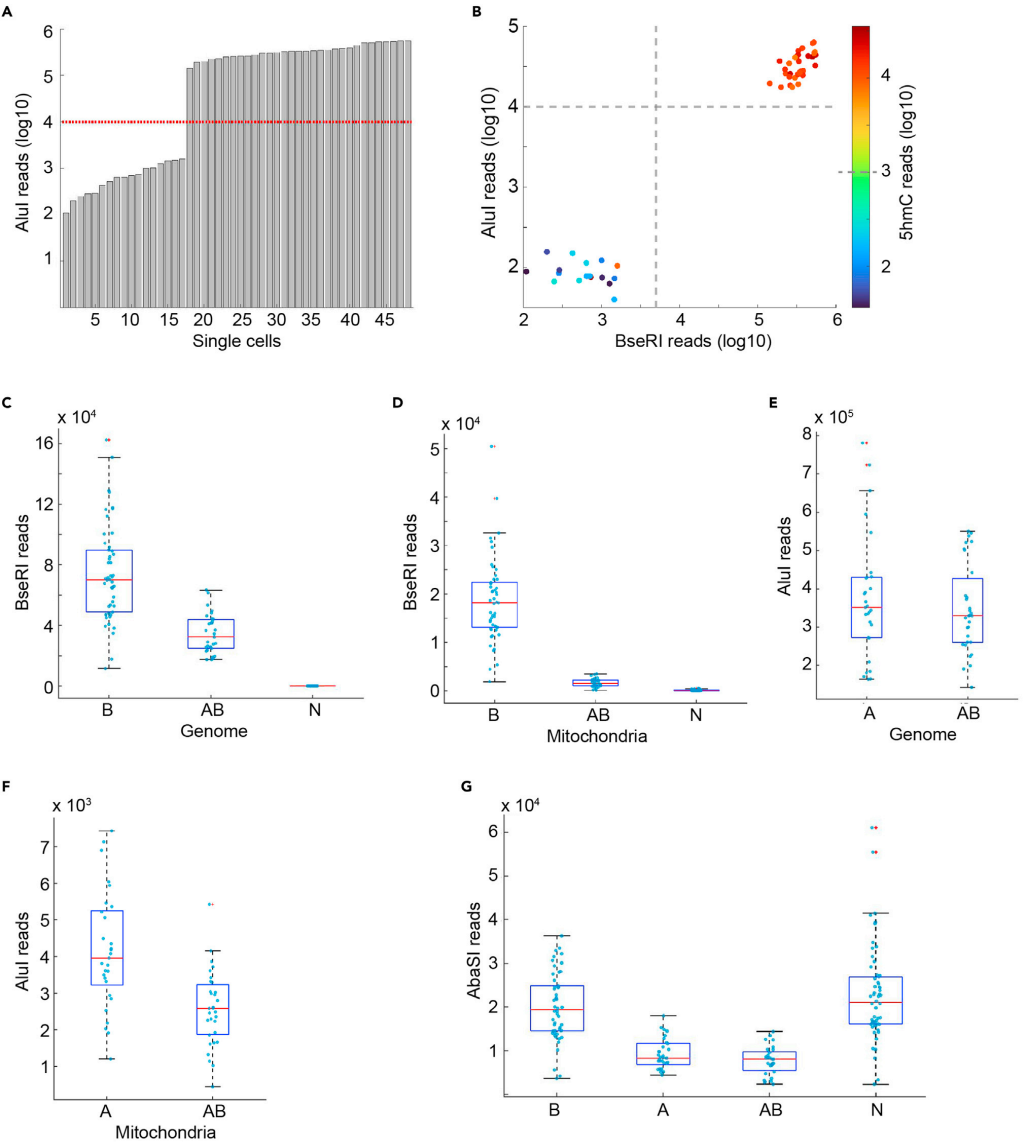
Expected sequencing metrics in scH&G-seq (A) The number of AluI-derived reads detected in successfully sequenced cells is several orders-of-magnitude higher than poorly sequenced cells. The red dotted line shows a cutoff between successfully and poorly sequenced cells. (B) When AbaSI, BseRI, and AluI are used, high counts are simultaneously detected for reads that derive from all three enzymes in successfully sequenced cells. The grey dotted lines show a cutoff between successfully and poorly sequenced cells. (C and D) Using either BseRI or a combination of AluI and BseRI enables the detection of BseRI-derived reads from genomic DNA (C) and mitochondrial DNA (D) compared to control. (E and F) Using either AluI or a combination of AluI and BseRI enables the detection of AluI-derived reads from genomic DNA (E) and mitochondrial DNA (F). As cells in the control sample received no AluI adapter, it has no associated AluI-derived reads, and is therefore not shown in these two panels. (G) Using BseRI, AluI, or AluI and BseRI, in combination with AbaSI results in the detection of 5hmC sites in the genome. “*A*”, “*B*”, “*AB*”, and “*N*” denote libraries made using AluI, BseRI, AluI and BseRI, and neither AluI and BseRI, respectively. All conditions shown here include the use of AbaSI for the detection of 5hmC.

Prior to sequencing, the bioanalyzer plot should show a size distribution that contains greater than 90% of the Illumina library between 200 and 1000 bp. There should be little to no primer (<100 bases) or dimer peaks (∼120 bases). The bioanalyzer plot for the Illumina library should be similar to that shown in Figure 3C. The concentration of the Illumina library can vary but it should be high enough to be detected on the Qubit dsDNA high-sensitivity assay with 1 μL of sample. Successfully sequenced cells typically result in several orders of magnitude more sequencing reads compared to barcodes that were not used (negative controls), or cells that were poorly sorted (Figure 4A). Nearly all cells that have high read counts derived from one enzymatic digestion also have high read counts from the other enzymatic digestion(s) (Figure 4B).

In the example shown in Figure 4, scH&G-seq was performed using BseRI (B), AluI (A), both enzymes (AB), or neither enzyme (N), along with AbaSI. As expected, genomic and mitochondrial BseRI-derived reads were detected in conditions B and AB (Figures 4C and 4D). Similarly, genomic and mitochondrial AluI-derived reads were detected in conditions A and AB (Figures 4E and 4F). In all conditions, high levels of AbaSI-derived reads were identified, demonstrating that in all these conditions 5hmC can be detected (Figure 4G).

## Quantification and statistical analysis

Strand-specific 5hmC can be used to infer cellular lineages at an individual cell division resolution. Further, sequencing of genomic and mitochondrial DNA can be used to identify copy number variations (CNV) and single-nucleotide polymorphisms (SNP) that together with strand-specific 5hmC can be used to reconstruct larger lineage trees. The detailed methods can be found in Wangsanuwat et al., 2021.

## Limitations

Certain cell types contain low levels of 5hmC and in these cases successful detection of this mark may be challenging in single cells. However, for applications related to reconstructing lineage trees, it is only necessary to quantify the relative levels of 5hmC between the two strands of a chromosome; therefore, in these cases, the absolute levels of 5hmC in a cell does not impact the accuracy of lineage reconstruction (Mooijman et al., 2016; Wangsanuwat et al., 2021).

## Troubleshooting

### Problem 1

Prior to IVT, the sample does not reduce to a volume of 6.4 μL, even after several minutes in the vacufuge (step 26).

### Potential solution

This problem most likely occurs because the sample contains a layer of oil, reducing the efficiency of evaporation. Return the sample to the vacufuge, and spin for an additional 10 min at 20°C–25°C. If no evaporation occurs, use the 30°C temperature mode on the vacufuge and continue spinning for an additional 10 min. If this fails, transfer the liquid to a new 1.5 μL microcentrifuge tube and continue spinning at 30°C. If necessary, a short vacufuge spin of less than 10 min at a temperature of 45°C can also be attempted. If the issue persists, continue with the protocol by proportionally scaling up all reagents of the IVT reaction, aRNA fragmentation and the aRNA bead cleanup. IVT has been successfully performed with an initial volume as high as 10 μL.

### Problem 2

After running the Agilent high-sensitivity DNA kit on the bioanalyzer, a significant amount of material and/or peaks are observed at sizes less than 200 bp (step 37).

### Potential solution

Perform an additional 0.825× AMPure XP magnetic bead cleanup and elute in 15 μL of nuclease-free water. Then rerun the Illumina library on the bioanalyzer using the Agilent high-sensitivity DNA kit.

### Problem 3

After running the Agilent high-sensitivity DNA kit on the bioanalyzer, a significant amount of material is observed at sizes greater than 1000 bp (step 37).

### Potential solution

Starting from the 5.25 μL of saved reverse transcribed aRNA (step 33.a), perform PCR with 2–3 less cycles. Continue to follow the protocol and re-evaluate the Illumina library on the bioanalyzer using the Agilent high-sensitivity DNA kit.

### Problem 4

After running the Agilent high-sensitivity DNA kit on the bioanalyzer, a very limited amount of material or no material is observed (step 37).

### Potential solution

Starting from the 5.25 μL of saved reverse transcribed aRNA (step 33.a), perform PCR with 3 more cycles. Continue to follow the protocol and re-evaluate the Illumina library on the bioanalyzer using the Agilent high-sensitivity DNA kit. If there is still a very limited amount of material, run an Agilent RNA 6000 Pico chip on the aRNA following the manufacturer’s recommendations to determine if the ligated genome DNA molecules were successfully amplified by IVT. If no material is observed on the Agilent RNA 6000 Pico chip, restart the protocol by processing a new plate (starting from step 10).

### Problem 5

Continued issues with generating high-quality Illumina libraries based on the bioanalyzer plots and the previous troubleshooting steps have not worked after multiple attempts (step 37).

### Potential solution

If none of the plates from a given FACS sort have been verified to work, restart by sorting single cells again into 384-well plates (starting from step 1). Replace reagents with new nuclease-free aliquots. If issues persist, lower the double-stranded adapter concentration by a factor of two. Lower double-stranded adapter concentrations typically produce high-quality Illumina libraries on the bioanalyzer at the expense of sequencing complexity. If issues persist, try sorting 100 cells into each well of a 384-well plate and perform scH&G-seq as described above to determine if single-cell sorting and/or handling of limited starting material is the problem.

## Resource availability

### Lead contact

Further information and requests for resources and reagents should be directed to and will be fulfilled by the lead contact, Siddharth S. Dey (sdey@ucsb.edu).

### Materials availability

This study did not generate new unique materials or reagents.

## Data Availability

The raw and processed single-cell sequencing data have been deposited at GEO and are publicly available as of the date of publication. The accession number is listed in the key resources table. The codes required to analyze sequencing data produced in this protocol have been deposited at GitHub and are publicly available as of the date of publication. The GitHub link is listed in the key resources table.
